# Quality of life in midwives after post-traumatic stress disorders

**DOI:** 10.1192/j.eurpsy.2022.1734

**Published:** 2022-09-01

**Authors:** I. Sellami, A. Feki, R. Masmoudi, K. Hammami, J. Masmoudi, M.L. Masmoudi, M. Hajjaji

**Affiliations:** 1 Hedi Chaker university hospital, Occupational Medicine, Sfax, Tunisia; 2 medecine university of Sfax, Rheumatology, Sfax, Tunisia; 3 HEDI CHAKER hospital, Psychiatry Department, SFAX, Tunisia; 4 CHU Hedi CHaker hospital Sfax Tunisia, Department Of Psychiatry (a), Sfax, Tunisia

**Keywords:** post traumatic stress disorder, midwives, Quality of Life

## Abstract

**Introduction:**

Post-traumatic stress disorder frequently alters the quality of life.

**Objectives:**

Assess the quality of life in midwives who have post-traumatic stress disorder.

**Methods:**

We conducted a cross-sectional study among midwives in a single university hospital centre using a self-administered questionnaire. We screened post-traumatic stress disorder using the Impact of event scale and the quality of life using 5 items Likert scale.

**Results:**

Our response rate was 82%. Out of 42 midwives who answered us, 18 had post-traumatic stress disorder symptoms (42.8%). They were all female. Their mean age was 45.6± 10.3 years. The traumatic event occurred mainly at work and was related to the death of a mother or a baby. Symptoms of post-traumatic stress disorder symptoms were severe in 5 midwives. The quality of life was altered in 38.8% of participants. Both post-traumatic stress disorder symptoms and alteration of the quality of life were more frequent in patients who don’t have leisure activities.

**Conclusions:**

In conclusion, midwives are vulnerable to developing post-traumatic stress disorder. Encouraging sports and other leisure activities may protect them from having severe repercussions on their life.

**Disclosure:**

No significant relationships.

